# Effects of Acute and Subchronic Anodal Transcranial Direct Current Stimulation (tDCS) on Morphine-Induced Responses in Hotplate Apparatus

**DOI:** 10.31661/gmj.v8i0.1157

**Published:** 2019-06-16

**Authors:** Seyyed Saeid Anvari, Mohammad Nasehi, Mohammad-Reza Zarrindast

**Affiliations:** ^1^Institute for Cognitive Science Studies (ICSS), Tehran, Iran; ^2^Cognitive and Neuroscience Research Center (CNRC), Tehran Medical Sciences, Islamic Azad University, Tehran, Iran; ^3^Department of Pharmacology, School of Medicine, Tehran University of Medical Sciences, Tehran, Iran

**Keywords:** tDCS, Prefrontal Cortex, Morphine, Pain, Anodal

## Abstract

**Background:**

The endogenous opioid system plays a basic role in pain suppression. The opiate analgesia is the most powerful and useful technique for reducing severe pain in many medical conditions. Transcranial direct current stimulation (tDCS) is a neuromodulator technique by which the cerebral cortex is stimulated with a weak and constant electrical current by the painless and non-invasive method.

**Materials and Methods:**

In this experimental study, we investigated the effect of tDCS on morphine (1.25, 2.5 and 5 mg/kg)-induced pain responses; as we applied left prefrontal anodal stimulation with 0.2 mA intensity and 20 minutes.

**Results:**

our results revealed that the acute (One-time electrical stimulation 24 hours after the last administration of morphine three days) and subchronic (three times electrical stimulation; one session/day before each administration of morphine three days) left prefrontal anodal tDCS does not alter pain perception induced by different dose of morphine significantly.

**Conclusion:**

Finally, our data indicated that there is no potentiated effect between acute tDCS or subchronic tDCS and morphine administration with tested parameters significantly.

## Introduction

The perception of pain is dynamically regulated by pain inhibitory and facilitatory pain circuits. Activation of these circuits terminates in the secretion of regulatory compounds that cause either pain suppression (analgesia) or pain facilitation (hyperalgesia), especially within the dorsal horn of the spinal cord. The endogenous opioid system plays a basic role in pain suppression; this system can also be activated by the administration of exogenous opiate drugs. Opiate analgesia is the most powerful and useful technique for reducing severe pain in many medical conditions [1]. Morphine is a µ-opioid analgesic applied in the management of moderate-to-severe cancer and postoperative pain. The µ-receptors expressed in the central nervous system (CNS) are responsible for supraspinal analgesia, respiratory depression, and sedation [2]. One of the most appropriate behavioral animal models for the evaluation of the pain response is hot plate test which is commonly employed to assess the effects of analgesic drugs that usually trigger changes in the nociceptive threshold. During a hot plate situation, rats display several noxious-evoked patterns (as hindpaw-licking, hindleg-withdrawal, jumping off, stamping) as well as exploratory and self-care responses that should be selected for quantifying the nociceptive threshold. Hindpaw-licking latency is usually used to evaluate it [3]. Brain transcranial direct current stimulation (tDCS) is a neuromodulator technique by which the cerebral cortex is stimulated with a weak and constant electrical current on the basis of the painless and non-invasive method. Mechanism of tDCS effect is not well understood, but preliminary studies using direct current applied on cerebral cortex in animals have shown that the anodal stimulation causes membrane depolarization and increasing the firing rate of cortical neurons in the under the context of electrode [4]. In a pilot study, tDCS seems to be safe, has minimal side effects, and may reduce post-procedural analgesia requirements and subjective pain ratings [5].In a previous study, it was observed that the antinociceptive effects of anodal-tDCS over motor cortex depend on different parameters. First, it was discovered that repetitive anodal-tDCS had a longer analgesic effect than single stimulus, and both ipsilateral-tDCS and contralateral-tDCS generate a long-lasting analgesic effect on neuropathic pain. Second, the antinociceptive effects were intensity-dependent and time-dependent, high intensities operated better than low intensities and long stimulus durations operated better than short stimulus durations. Third, the timing of the intervention after injury changed the stimulation outcome, timely use of tDCS was an effective manner to prevent the extension of pain, and more frequent intervention causes more analgesia in chronic constriction injury rats, finally, similar antinociceptive effects of contralateral-tDCS and ipsilateral-tDCS were considered in both sexes of rats [6]. In other study, findings indicated preliminary evidence that the analgesic effects reported with primary motor cortex(M1)-tDCS, can be in part associated with the recruitment of the same endogenous mu-opioid receptor mechanisms induced by placebo, and that such effects can be purposely optimized by real tDCS [7]. As well as findings represented that the analgesic effect of viewing the body was enhanced selectively by anodal stimulation of the occipital cortex. The effect was specific for the polarity and the site of stimulation. The results indicate that visually induced analgesia may associate with neural signals from the extrastriate visual cortex [8].There is evidence indicating that patients with chronic migraine have a positive, but delayed response to anodal tDCS of the primary motor cortex. These effects may be associated with electrical currents produced in pain-related cortical and subcortical regions [9]. A pilot trial was the first study to represent an opioid-sparing effect of tDCS after spine surgical procedures [10]. The dorsolateral prefrontal cortex (DLPFC) has been a main target of non-invasive stimulation techniques, such as tDCS. This brain region is easily accessible to stimulation and based on some results, anodal tDCS over the left DLPFC seems to act in a selective manner and would improve specific symptoms, particularly neuropathic pain [11]. Also, results of another study suggest that anodal tDCS over the left prefrontal cortex may be a suitable approach for decreasing post-total knee arthroplasty opioid requirements [12]. Although in a similar study have reported that four sessions of tDCS over motor cortex could decrease morphine consumption and pain perception during the postoperative period in total knee arthroplasty [13]. Regarding the importance of the topic of pain and its optimal control with the minimal amount of opioids to prevent drug tolerance, this study aimed to determine the effect of acute and subchronic anodal left prefrontal tDCS on morphine-induced pain perception in male Wistar rats.

## Materials and methods

### 1. Animals

Subjects in this experimental study were 96 male Wistar rats weighing 200-220 g, obtained from the animal house of the Institute for Cognitive Science Studies (ICSS); Tehran- Iran. Animals were divided into 12 Groups (n=8 in each group). The study procedures were performed under certain laboratory conditions (room temperature 22 ± 2°C with the light-dark cycle of 12 hours light and 12 hours darkness; lights turned on at 7:00 A.M.); also animals had free access to food and water. Each rat was only used once in the test-pretest protocol. Behavioral tests were also performed in the process of lighting. The animals were randomly allocated to the experimental groups and the experimental procedures were done in accordance with the National Institutes of Health Guide for the Care and Use of Laboratory Animals (NIH publications No. 80–23).

### 2. Drugs

During the experiments, morphine sulfate was dissolved daily in sterile 0.9% saline and prepared at different concentrations 1.25, 2.5 and 5 mg/kg for subcutaneous injection according to previous studies [14, 15].

### 3. Stereotaxic Surgery and Electrode Implantation

The animals received general anesthesia using an intraperitoneal injection of ketamine 10% (50mg/kg) and xylazine 2% (5 mg/kg) and then placed in stereotaxic equipment. Anodal electrode in the form of a tubular electrode with an internal diameter 2.1 mm and contact area 3.5 mm^2^ [16, 17] was positioned over the left prefrontal cortex 1.5 mm anterior to the coronal fissure and 1.5 mm left of the sagittal fissure according to the atlas of Paxinos and Watson( 1986) and then fixed with a coating of dental cement. After surgery, all animals were given a 4-7 days recovery period before receiving tDCS. During this period as well as during the electrical stimulation, rats were put pairs in each cage.

### 4. tDCS

The tDCS consists of a current generator and two electrodes [18]. The anodal electrode in the form of a tubular electrode was filled with saline solution (NaCl 0.9%) before the stimulation to create a contact area of 3.5mm^2^on the skull. Also, cathodal electrode (9.5 cm^2^sponge-covered rubber-plate electrode soaked in saline solution is placed onto the ventral thorax) into the hand-made jacket operated as the counter-electrode. This setting allowed us to prevent electrical current diversion and to protect the safety and efficiency of the stimulation [19, 16]. Active Dose ΙΙ unit (Activatek Company-Taiwan) was utilized as tDCS for electrical stimulation. However, all groups were stimulated with an anodal 20 min/day constant current of 0.2 mA was used transcranially over the left prefrontal cortex for one day or three consecutive days. Control groups were obtained sham tDCS. In order to prevent probabilistic interactions between tDCS effects and anesthetic drugs, animals were awake and unrestrained in a cage during the tDCS.

### 5. Hot Plate Test

For evaluation of variations in pain threshold, individual rats were placed on the hot plate (BorjSanatAzma Co, Tehran, Iran), while the temperature of apparatus maintained at 50 ± 1°C during the test. This apparatus contains a rectangular cast-iron plate (20×25) that is provided with a thermostat, power supply, and a holding cylinder (with a diagonal of 20 cm and height of 30 cm). Licking one of the hind paws or first jumping was recorded as an index of pain reaction latency in seconds, while the cut-off time of the test was 60 seconds for rats that did not respond [20-22]. The test was applied for each rat in 24 hours after the last session of subchronic tDCS or 20 min after the session of acute tDCS.

### 6. Experimental Protocols

#### 6.1. Dose-Response Effects of Morphine-Induced Variation in Pain Perception

In this experiment, four groups (n=8 in each group) of rats were applied. Three groups of rats obtained saline (10 ml/kg, s.c.) or different doses of morphine (1.25, 2.5 and 5 mg/kg, s.c.) during three consecutive days. Rats were tested by the hot plate 24 hours after the last injection.

#### 6.2. Effects of Subchronic Anodal tDCS On the Morphine-Induced Responses in Hotplate Test

In this experiment, four groups of rats (n=8 in each group), anodal tDCS on the left prefrontal were received 3 sessions during three consecutive days just before the administration of saline (10 ml/kg, s.c.) or different doses of morphine (1.25, 2.5 and 5 mg/kg, s.c.). Rats were tested by the hot plate 24 hours after the last session.

#### 6.3. Effects of Acute Anodal tDCS On the Morphine-Induced Responses in Hotplate Test

In this experiment, four groups of rats (n=8 in each group), anodal tDCS on the left prefrontal were received 1 session 24 hours after the administration three consecutive days of saline (10 ml/kg, s.c.) or different doses of morphine (1.25, 2.5 and 5 mg/kg, s.c.) and just before the hot plate test. Rats were tested by the hot plate 20 min after the session.

### 7. Statistical Analysis

SPSS version 16 software (IBM company, New York, U.S.A.) was applied for data analysis. The between-group comparisons were performed with one or two-way analysis of variance (ANOVA) tests. Following significant ANOVA results, post hoc analysis (Turkey’s test) was used for inter-group comparisons. The significance level was considered at P ≤ 0.05 for all the statistical comparisons. Finally, Sigma plot version 12 software (Systat Software Inc., San Jose, California) was applied to draw figures.

## Results

### Dose-Response Effects of Morphine-Induced Variation in Pain Perception

**Figure 1:**
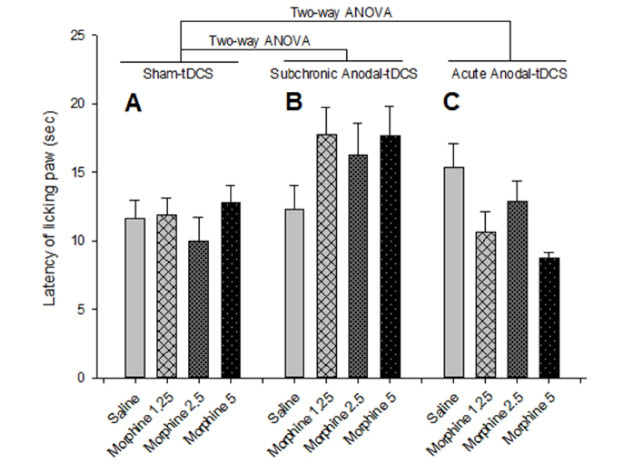
The effects of left prefrontal anodal tDCS on the morphine-induced responses in hotplate apparatus. Latency as time of first reaction to thermal pain in the form of licking paws or special alterations in rats' steps was recorded based on seconds. Result is expressed as mean ± S.E.M of 8 rats per group. A: Dose-Response effects of morphine-induced pain perception. B: The effects of subchronic anodal tDCS on the morphine-induced responses in hotplate apparatus. C: The effects of acute anodal tDCS on the morphine-induced responses in hotplate apparatus.

Figure-1A shows that administration of saline (10 ml/kg, s.c.) and different doses of morphine (1.25, 2.5 and 5 mg/kg, s.c.) during three consecutive days does not significantly alter the pain perception in different doses on the day after last morphine injection.

### Effects of Subchronic Anodal tDCS On the Morphine-Induced Responses in Hotplate Test

According to the Figure-1B, two-way ANOVA indicated that there is no significant interaction between tDCS and morphine in pain perception after 24 hours following subchronic anodal left prefrontal tDCS while tDCS effect was significant alone [within group comparison: for Drug effect, F(3, 56)=1.453, P=0.237; tDCS effect, F(1, 56)=12.315, P=0.001; and Drug × tDCS interaction effect, F(3, 56)=1.061, P=0.373].

### Effects of Acute Anodal tDCS On the Morphine-Induced Responses in Hot Plate Test

According to the Figure-1C, Two-way ANOVA indicated a significant interaction between tDCS and morphine in pain perception after 24 hours following acute anodal left prefrontal tDCS [within group comparison: for Drug effect, F(3, 56)=1.516, P=0.220; tDCS effect, F(1, 56)=0.122, P=0.728; and Drug × tDCS interaction effect, F(3, 56)=3.389, P=0.024]. Although, post hoc analysis represented that the pre-test acute anodal left prefrontal tDCS does not significantly alter the analgesic effect in different doses on the day after the last morphine injection.

## Discussion

In the present study, we did not observe any significant responses in hotplate test 24 hours after the last morphine injection (3 consecutive days) in different doses. According to a previous study that represented in the mole-rat, opioid systems in the CNS may not be involved in the regulation of analgesia, but it can regulate several activity such as motor activity [23]. As well as, the other results of the hotplate test indicated that morphine (5 and 10 mg/kg) induced significant analgesia in naive rats but its analgesic effects in rats receiving 15 days injections of morphine (10 mg/kg) was reduced, showing tolerance to morphine analgesia [22]. The results of several studies have also indicated that NMDA receptor gene expression at mRNA level in rats tolerant to morphine is significantly increased in the striatum but decreased in the PFC. Therefore; variations in the gene expression in rat striatum and PFC have a region-specific association with morphine-induced analgesic tolerance [24]. In addition, the other results indicated that glutamate receptor gene expression in tolerant rats was reduced in the lumbosacral cord but enhanced in the midbrain compared to the control group. However, no significant variation was considered for mu-opioid receptor gene expression in both areas [22].In order to evaluate the effect of tDCS on the morphine-induced responses in hotplate test, we transcranially applied anodal stimulation over the left prefrontal cortex with the duration of 20 min/day and constant current of 0.2 mA for one day or three consecutive days. In order to evaluate effect of subchronic tDCS on morphine-induced responses in hotplate test, our results revealed that applying left prefrontal subchronic anodal tDCS during 3 sessions (three consecutive days) just before the morphine administration in different doses, does not significantly increase responses in hotplate test in different doses of morphine 24 hours after the last session. Also, to assess the effect of acute tDCS on morphine-induced responses in hotplate test, our results showed that applying left prefrontal acute anodal tDCS during 1 session at 24 hours after last morphine injection (3 consecutive days) in different doses, despite of indicating a significant interaction between tDCS and morphine, does not alter responses in hotplate test. The results of an investigation demonstrated that both M1 and DLFPC anodal tDCS could be applied to regulated pain thresholds in healthy subjects [25]. According to the results of another study, left-DLPFC-tDCS induces an antinociceptive effect, which is explained by decreased perfusion to posterior insula and thalamus. Also, this proposes structural dependence by the neuromodulatory process to induce analgesia with potential relevance for patient stratification [26]. According to results of some studies, those indicated that long-term morphine administration creates tolerance to the antinociceptive effect of the opioid, as disclosed by a significant reduction in morphine-induced antinociceptive on day eight compared to day 1 of the injections [24]. Based on studies, several factors may describe the lack of objective impact of tDCS on morphine consumption and pain perception: the procedure of brain stimulation (tDCS/rTMS; repetitive Transcranial Magnetic Stimulation), possible interactions with anesthetic drugs, variations in subjects’ population, and the prior experience of pain and long-term utilization of antinociceptive drugs. Further studies with tDCS should be accomplished before deducing that tDCS is incompetent for postoperative pain management, because noninvasive brain stimulation procedures, such as rTMS and tDCS, may become absorbed in the modulation of several different modes of analgesia [27].

## Conclusion

Finally, our data indicated that there is no any potentiated effect between acute tDCS or subchronic tDCS and morphine administration with tested parameters significantly. However, changing the test parameters such as stimulation parameters, stimulation sessions, and evaluation interval of responses in the hotplate test may indicate the effective interactions between morphine and tDCS in the induction of analgesic effects.

## Acknowledgment

The authors would like to express their appreciation to Dr. Fariborz Manteghi for designing of the electrode.

## Conflict of Interest

The authors do not declare any conflict of interest related to this work.
